# High-resolution datasets for urban heat vulnerability assessment in urbanized areas of the Netherlands

**DOI:** 10.1016/j.dib.2025.111525

**Published:** 2025-03-28

**Authors:** Maha M. Habib, Marjolein van Esch, Maarten van Ham, Wim J. Timmermans

**Affiliations:** a Department of Urbanism, Faculty of Architecture and the Built Environment, Delft University of Technology, Julianalaan 134, 2628 BL, Delft, the Netherlands; b Department of Water Resources, Faculty of Geo-Information Science and Earth Observation, University of Twente, Hallenweg 8, 7522 NH, Enschede, the Netherlands

**Keywords:** Urban heat island, Urban climate, Sky view factor, Street canyon, Built environment

## Abstract

The urban heat island effect is increasingly affecting the quality of life in cities, and detailed data is crucial in designing mitigation policies. However, weather stations are predominantly situated outside urban environments, limiting their ability to represent the varying air temperatures within street canyons. This data paper addresses this limitation by presenting a dataset of the modeled daily maximum urban heat island (UHI_max_) effect across 99 Dutch municipalities during the summer of 2023. This is achieved by implementing a semi-empirical equation that incorporates readily available meteorological variables and two key urban morphological indicators, namely the sky view factor and fractional vegetation cover. Two primary datasets are presented: (1) a high-resolution dataset of modeled UHI_max_, and (2) a sky view factor dataset. Both datasets are provided in GeoTIFF format at a 5-meter spatial resolution. Additionally, this paper presents a straightforward methodology for obtaining UHI_max_ values for other periods. The datasets and accompanying methodology provide valuable resources for advancing urban climate research, urban planning and heat mitigation strategies in the Netherlands.

Specification Table

**Table:** 

Subject	Urban climatology
Specific subject area	Urban heat island
Type of data	GeoTIFF
Data collection	All data were derived from publicly available sources and links to secondary datasets are provided in the repository: • Digital Surface Model (DSM) from AHN4 (Actueel Hoogtebestand Nederland, version 4) at 0.5-meter resolution • Meteorological data and coordinates from KNMI (Royal Netherlands Meteorological Institute) weather stations • Fractional vegetation cover data from RIVM (National Institute for Public Health and the Environment) at 10-meter resolution, categorizing vegetation into trees, shrubs and grass • Building footprint geometry from 3DBAG (3D Building models Automatically Generated) • Administrative boundaries from CBS (Statistics Netherlands)
Data source location	Country: The Netherlands City: Data were collected and processed for 99 Dutch municipalities with a population density exceeding 1,000 people per km²
Data accessibility	Repository name: 4TU.ResearchData Data identification number: https://doi.org/10.4121/0159bc0e-3ab9-42b6-8b12-ad3b2294face Direct URL to data: https://data.4tu.nl/datasets/0159bc0e-3ab9-42b6-8b12-ad3b2294face
Related research article	none

## 1 Value of the Data

• The datasets support urban heat vulnerability assessments and analysis related to thermal comfort, public health, productivity and energy consumption.
• While the UHI_max_ dataset is compiled for a specific timeframe, this data paper adopts a methodology developed by [1] that leverages open-source tools and data to provide a flexible setup for researchers to generate UHI_max_ for different periods and conduct various temporal analyses.
• The application of the sky view factor extends beyond urban heat assessments, facilitating studies of various phenomena, including urban energy balance [2], noise propagation [3] and air pollution [4].
• The extensive spatial coverage of the UHI_max_ dataset enables cross-regional and international comparative studies, complementing existing datasets [5,6].

## 2 Background

Cities face increasing thermal challenges during summertime due to rising temperatures and the urban Hheat island (UHI) effect, which significantly affects the quality of life for urban residents. The UHI effect is largely caused by urbanized areas trapping more heat than their rural counterparts. Urban areas have more paved surfaces and materials that absorb and retain heat, structures that obstruct natural airflow, and fewer trees and plants that potentially provide cooling [7,8]. The UHI_max_ can be calculated by obtaining the maximum hourly difference between urban air temperature (T_urban_) and rural air temperature (T_rural_):

(1) $$UHI_{\text{max}}=\max\left(T_{urban}-T_{rural}\right)$$

Given that access to weather station measurements is possible, deriving UHI_max_ is relatively straightforward. However, most weather stations are located outside cities, often near airports, which results in limited air temperature data that is representative of urban canyons. While proprietary software packages like ENVI-met and SOLENE-microclimate can simulate air temperatures within street canyons, their application over extended periods and large spatial areas is constrained by scalability challenges [9]. To address these challenges, a semi-empirical equation has been proposed for estimating UHI_max_ in northwestern European cities [1]. This method leverages readily accessible data from rural weather stations and incorporates local urban indicators, namely the sky view factor (SVF) and fractional vegetation cover (VEG_f_), offering a more straightforward and scalable approach. Furthermore, the method enables the generation of high-resolution UHI_max_ datasets, which form the basis of this paper. These datasets, along with the supporting methodology, provide valuable resources for further research and practical applications in urban climatology and planning.

## 3 Data Description

**Fig. 1:**
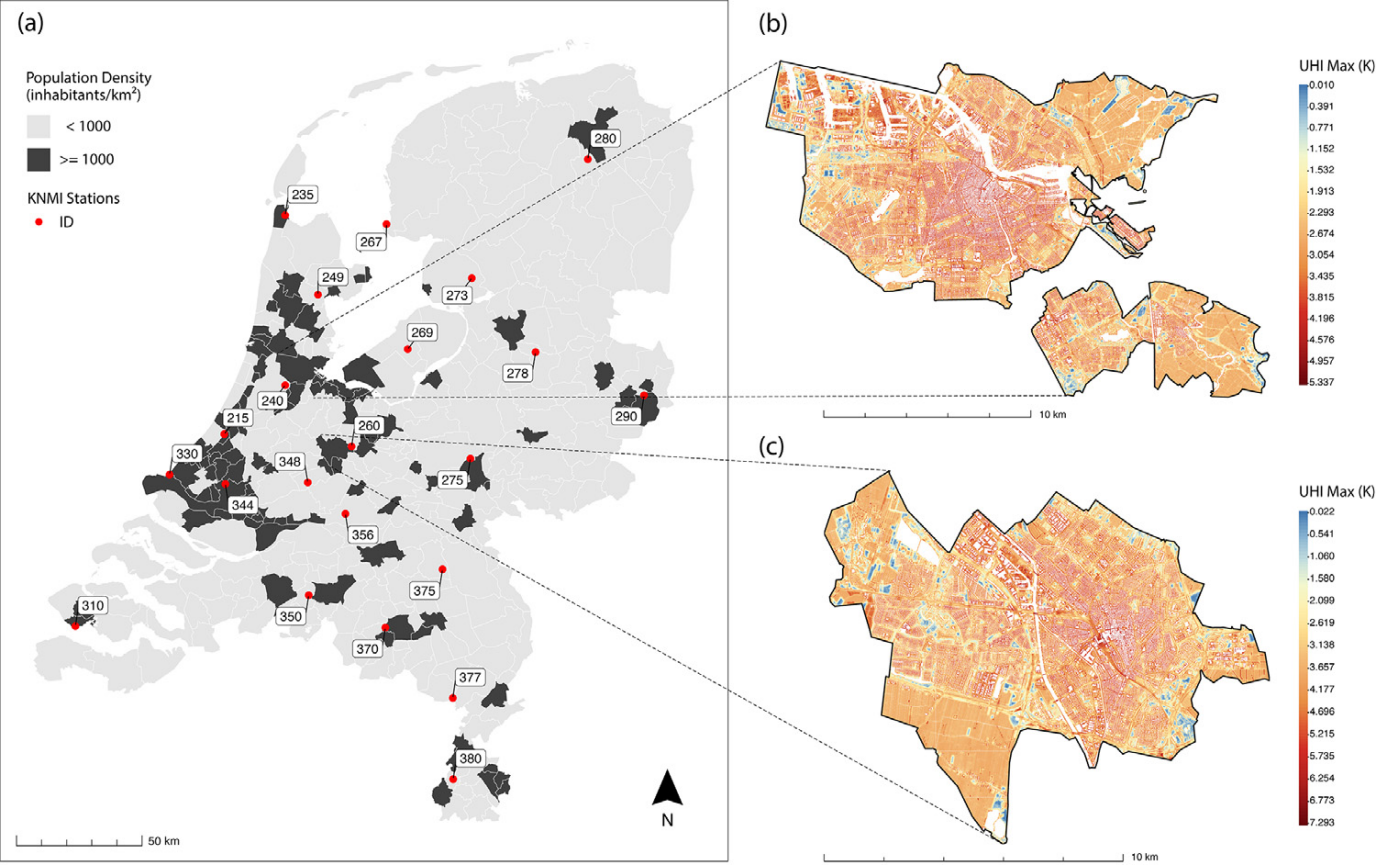
Spatial distribution of (a) Dutch municipalities with a population density of ≥1000 inhabitants/km² and their assigned rural weather stations, showing station ID numbers (red dots); (b) & (c) provide examples of UHI_max_ intensity occurrences for Amsterdam and Utrecht respectively on 2023-06-10, illustrating spatial temperature variations in Kelvin.

This data paper presents two primary datasets for the Netherlands that enable the assessment of urban heat vulnerability. Both datasets are derived from publicly available sources and processed at 5-meter resolution:

1. SVF: A GeoTIFF dataset quantifying sky visibility in street canyons, with values ranging from 0 (completely obstructed) to 1 (completely unobstructed). The dataset was derived from 375 Digital Surface Model (DSM) mosaics from AHN4, collected between 2020 and 2022.
2. UHI_max_: A collection of GeoTIFF files covering 99 urbanized Dutch municipalities, each capturing UHI_max_ for a specific occurrence during the summer period of 2023 (June 1 to September 30), measured in Kelvin (K). The specific UHI_max_ occurrence for each municipality is outlined in Table 1. These datasets were generated using an empirical equation that incorporates both local urban morphological indicators (SVF and VEG_f_) and meteorological measurements from nearby KNMI weather stations. Each municipality is paired with its nearest rural weather station to obtain the appropriate meteorological parameters for the UHI_max_ calculation. For each municipality, a weather station is assigned, and their geographical locations are presented in Table 1 and Fig. 1.

**Table 1:** Rural weather station locations and their associated municipalities for estimating UHI_max_ occurrence.

Station ID	Coordinates (X, Y)	Municipalities	UHI_max_ Date (YYYY-MM-DD)
215	(89.91, 461.71)	Katwijk, Leiden, Leiderdorp, Lisse, Oegstgeest, Voorschoten, Zoetermeer, Teylingen, Leidschendam-Voorburg, 's-Gravenhage	2023-06-14
235	(114.24, 549.05)	Den Helder	2023-06-13
240	(114.26, 481.20)	Aalsmeer, Amstelveen, Amsterdam, Beverwijk, Diemen, Haarlem, Heemskerk, Heemstede, Uithoorn, Velsen, Zaanstad, Hillegom, Gooise Meren	2023-06-10
249	(127.36, 517.33)	Alkmaar, Heiloo, Hoorn, Purmerend, Stede Broec, Dijk en Waard	2023-06-13
260	(140.78, 456.76)	Amersfoort, Soest, Zeist, Nieuwegein, Blaricum, Hilversum, Huizen, Utrecht	2023-06-10
267	(154.74, 545.51)	Enkhuizen	2023-06-13
269	(163.22, 495.62)	Almere, Harderwijk	2023-06-12
273	(188.82, 523.95)	Urk	2023-06-12
275	(188.27, 451.94)	Arnhem, Wageningen, Westervoort, Zutphen, Veenendaal	2023-06-12
278	(214.28, 494.43)	Zwolle	2023-06-12
280	(235.16, 571.43)	Groningen	2023-07-08
290	(257.62, 477.18)	Almelo, Enschede, Hengelo (O.), Oldenzaal	2023-07-08
310	(30.46, 385.12)	Middelburg (Z.), Vlissingen, Maassluis, Westland	2023-06-10
330	(68.08, 445.50)	Maassluis, Westland	2023-06-14
344	(90.38, 441.77)	Alblasserdam, Barendrecht, Capelle aan den IJssel, Delft, Hendrik-Ido-Ambacht, Krimpen aan den IJssel, Ridderkerk, Rotterdam, Rijswijk (ZH.), Schiedam, Albrandswaard, Vlaardingen, Waddinxveen, Zwijndrecht, Lansingerland, Pijnacker-Nootdorp, Nissewaard	2023-06-13
348	(123.30, 442.41)	IJsselstein, Dordrecht, Gouda, Hardinxveld-Giessendam, Papendrecht, Sliedrecht	2023-06-12
350	(123.66, 397.44)	Breda, Tilburg	2023-06-15
356	(138.33, 429.94)	Culemborg, Tiel, Gorinchem, 's -Hertogenbosch	2023-06-11
370	(154.29, 384.52)	Eindhoven, Helmond, Veldhoven, Geldrop-Mierlo	2023-06-15
375	(177.11, 407.80)	Nijmegen	2023-06-12
377	(181.23, 356.43)	Roermond	2023-06-12
380	(181.36, 324.01)	Landgraaf, Brunssum, Heerlen, Kerkrade, Maastricht, Stein (L.), Sittard-Geleen	2023-06-16

## 4 Experimental Design, Materials and Methods

Following [1], the UHI_max_ equation comprises two components: one representing input from a given urban area and the other incorporating meteorological variables from nearby rural weather stations. The equation is expressed as follows:

(2) $$UHI_{\text{max}}=\left(2-SVF-VEG_{f}\right)\sqrt[{4}]{(DTR^{3}\cdot S)/U}$$

Here:

• *SVF* represents the sky view factor, measured from a specific point at street level within the local urban area.
• *VEG_f_* represents the fractional vegetation cover, measured within the local urban area, averaged over a 250 meters radius.
• *DTR* represents diurnal temperature range, calculated as daily (*T*_max_* − T*_min_) measured in a rural area.
• *S* represents solar irradiance, calculated as the 24-hour average of hourly maximum shortwave incoming radiation measured in a rural area.
• *U* represents the 24-hour average of hourly wind speed, measured at 10 m above ground in a rural area.

**Fig. 2:**
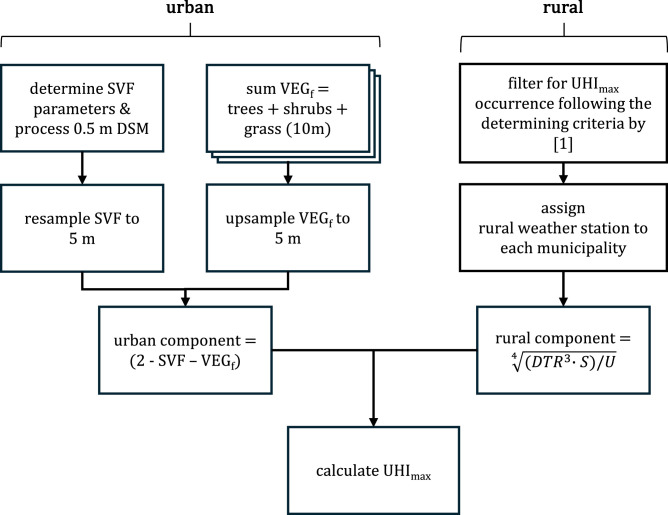
Flowchart outlining methodological process.

Fig. 2 outlines the workflow for this data paper. Details on the methodology, preprocessing steps and instructions for reproducing other occurrences of UHI_max_ are all provided in the accompanying README.md file available in the data repository on 4TU.ResearchData.

### 4.1 Calculating morphological indicators

#### 4.1.1 Sky view factor

**Fig. 3:**
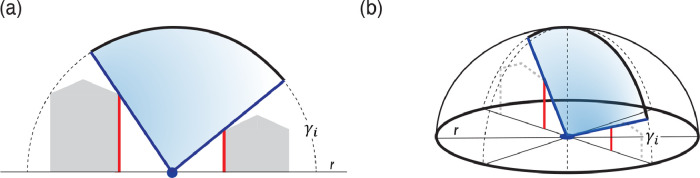
Calculation of SVF: (a) cross-sectional view of a street canyon illustrating the measurement of a single horizon elevation angle ($\gamma_{i}$) between buildings. The angle is measured relative to the street level; (b) three-dimensional hemispheric projection showing the sampling of elevation angles ($\gamma_{i}$) in n radial directions within a defined search radius (r).

A key urban morphological indicator is the SVF, which influences the surface radiation balance by quantifying how much of the sky is visible from a given point [1,10], as shown in Fig. 3. The SVF ranges from 0 to 1 and is determined based on a defined search radius and the number of directions considered to account for obstructions in the built environment. Values close to 1 indicate that nearly the entire sky hemisphere is visible, typical of open areas such as wide streets or plazas. In contrast, values close to 0 are observed in enclosed areas, such as narrow streets or deep urban canyons, where the sky is largely obstructed by surrounding buildings.

The SVF dataset was generated by combining multiple high-resolution DSM mosaics with a resolution of 0.5 m. To keep file sizes manageable, the final SVF output was resampled to a 5 m resolution. A total of 375 DSM tiles, each covering an area of 6.25 × 5 km, were used in the computation. These tiles were clustered and clipped using a moving window method. The processing was implemented in Python and R, utilizing a method developed by [11], which expresses the SVF as:

(3) $$SVF=1-\left(\sum_{i=1}^{n}\sin\gamma_{i}\right)/\;n$$

Following the calculation of SVF, the building footprints were masked since SVF values are only meaningful in street canyons [12], where they represent the amount of visible sky affecting outdoor thermal conditions.

#### 4.1.2 Sky view factor sensitivity analysis

**Fig. 4:**
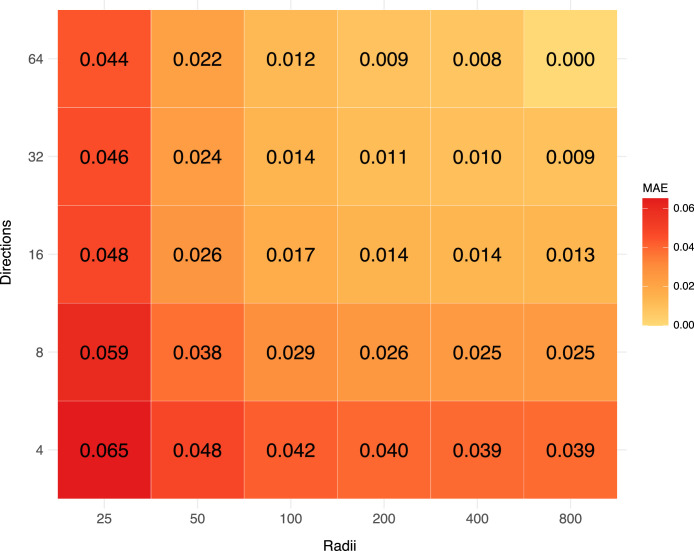
MAE of SVF calculations for Rotterdam, comparing search radii (25–800 m) and sampling directions (4–64) at 1 m resolution.

Computing Eq. (3) demands significant computational resources. To balance computational efficiency and accuracy, a sensitivity analysis was conducted to identify the optimal parameters for the final SVF calculations. Using Rotterdam—a city with diverse urban morphology—as a test case, the analysis evaluated how varying search radii (ranging from 25 m to 800 m) and directional sampling densities (ranging from 4 to 64 directions) influenced the results, as shown in Fig. 4.

The sensitivity analysis revealed that increasing the search radius beyond 100 m or the number of directions beyond 16 resulted in minimal improvement in the absolute mean error (MAE). The MAE was determined by comparing the results to a baseline with a search radius of 800 m and 64 directions, which yielded an MAE of 0. These findings are consistent with those of [12], who conducted a similar study in Utrecht. As a result, a search radius of 100 m and 16 directions were selected for the SVF calculations.

Despite these optimizations, the computational process remained resource-intensive. To manage the workflow efficiently, the DelftBlue supercomputer was used [13].

#### 4.1.3 Fractional vegetation cover

The VEG_f_ calculation followed the method outlined in [12], which utilizes publicly available vegetation data for the Netherlands at 10-meter resolution. This dataset classifies vegetation into three height-based categories: trees (>2.5 m), shrubs (1.0–2.5 m) and low vegetation (<1.0 m). Each pixel in the dataset represents the percentage coverage of each vegetation type, with the three categories collectively summing to a maximum of 100% for any given pixel. Buildings, agricultural land and other non-urban land cover types were excluded from the analysis.

VEG_f_ values were calculated by summing the three vegetation categories. The total VEG_f_ was then normalized to a scale from 0 to 1. To align with the SVF dataset, the final GeoTIFF was resampled to a 5-meter resolution and averaged over a 250-meter radius for each SVF pixel. Finally, for each municipality, the first part of Eq. (2) was calculated by subtracting SVF and VEG_f_ from the constant to determine their contribution to the equation.

### 4.2 Meteorological variables

The second component of Eq. (2) involves deriving hourly measurements from rural weather stations. Fig. 1 displays the 31 KNMI weather stations used in this study. To identify UHI_max_ occurrences, fair weather conditions are required. Following the methodology described in [1], meteorological data was filtered to exclude conditions that suppress UHI effects. Specifically, days were omitted if frontal systems (boundaries between different air masses) were present, precipitation exceeded 0.3 mm, hourly average wind speeds between successive hours differed by more than 2 ms⁻¹ per hour or relative humidity exceeded 80%.

Once these criteria were met, each municipality was assigned the nearest KNMI weather station along with its corresponding rural measurements—DTR, S and U. These measurements were incorporated into Eq. (2) to estimate UHI_max_ for each municipality. After incorporating the urban component, the occurrence of UHI_max_ were determined for the summer of 2023, spanning June to September. While this dataset focuses on a specific occurrence of UHI_max_, the methodology is adaptable, given that hourly weather data and updated vegetation cover information are available.

## Limitations

**Fig. 5:**
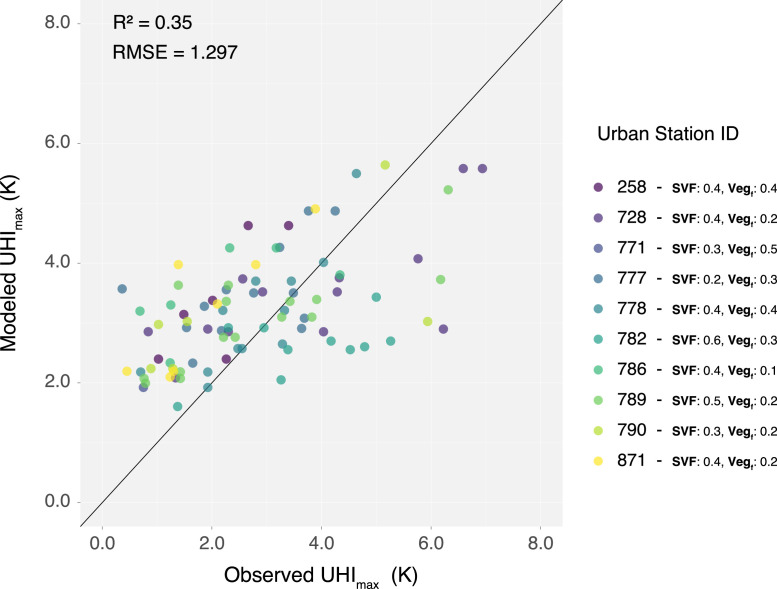
Validation of UHI_max_ model against observed measurements from urban stations in Utrecht. All urban stations have SVF values ranging from 0.2–0.6 and vegetational cover ranging from 0.1–0.5 measured within a 250 m radius.

The presented datasets have two limitations. First, the SVF dataset has imprecise edge estimates due to insufficient neighboring data. This issue arises because calculating SVF values requires 100 m search radius, and at the dataset's edges, there is insufficient data beyond the boundary to complete these calculations. Therefore, it is recommended to clip the dataset by 100 m at the boundaries. Second, validation of the UHI_max_ dataset was performed using Eq. (1), yielding an RMSE of 1.29 K, compared to an RMSE of 0.91 K reported by [1] (Fig. 5). Errors of approximately 1–2 K are anticipated due to the significant uncertainties inherent in urban climate predictions [9,14]. The diagnostic equation does not account for several urban-related variables, such as building materials and anthropogenic heat release, due to uncertainties in the available estimation methods. It is suggested by [1] that if the anthropogenic heat release is known, to incorporate it into the equation. Another reason for the systematic difference may be attributed to the validation process, which utilized temperature data from Utrecht, collected by sensors from a citizen science initiative [15]. The sensors were carefully selected to minimize data quality issues. This was achieved by evaluating GPS consistency, ensuring that more than 80% of data packets were successfully transmitted and received. Additionally, the temperature time series for each station was visually inspected to confirm continuous measurement patterns. Stations were excluded if their temperature readings exhibited irregular patterns, such as abrupt fluctuations inconsistent with expected trends or prolonged data gaps. These gaps, observed in the plotted temperature data, indicated incomplete or unreliable measurements. Despite this careful selection, challenges remained. The analysis revealed considerable sensor movement over time, with locations shifting by up to 1.5 km, which is particularly problematic for accurately determining maximum urban temperatures.

## Ethics Statement

The authors have read and comply with the ethical requirements for publication in Data in Brief. They also confirm that this work does not involve human subjects, animal experiments, or any data collected from social media platforms.

## CRediT Author Statement

**Maha M. Habib:** Conceptualization, Methodology, Data Curation, Original Draft Preparation, Formal Analysis, Investigation, Writing and Validation. **Marjolein van Esch:** Supervision, Reviewing and Editing. **Maarten van Ham:** Project Administration, Reviewing and Editing. **Wim J. Timmermans:** Supervision, Reviewing and Editing.

## Data Availability

4TU.ResearchDataHigh-Resolution Datasets for Heat Vulnerability Assessment in High-Density Urban Areas of the Netherlands (Original data).
